# Resection of thoracic malignancies infiltrating cardiac structures with use of cardiopulmonary bypass

**DOI:** 10.1186/s13019-015-0296-8

**Published:** 2015-06-25

**Authors:** Rawa Arif, Florian Eichhorn, Klaus Kallenbach, Philipp Seppelt, Arjang Ruhparwar, Hendrik Dienemann, Matthias Karck

**Affiliations:** 1 Department of Cardiac Surgery, University Hospital Heidelberg, Im Neuenheimer Feld 110, 69120 Heidelberg, Germany; 2 Department of Thoracic Surgery, Thoraxklinik Heidelberg, University Hospital Heidelberg, Heidelberg, Germany

**Keywords:** Tumor, Sarcoma, Mediastinal tumor, Metastases, Thymus

## Abstract

**Background:**

Only few reports exist on malignant thoracic neoplasms that require cardiopulmonary bypass during resection. We aimed to investigate the early and late clinical outcome of these patients.

**Methods:**

Patients with thoracic malignancies that underwent surgery between 2002 and 2014 were analyzed. All patients had cardiopulomonary bypass support during resection. Clinical and perioperative data was retrospectively reviewed for outcome and overall survival.

**Results:**

Fifteen patients (12 female, mean age of 55 ± 15 years, range 24 to 80 years) were identified. Eleven (8 female) were diagnosed with primary thoracic malignomas and four with metastases. Three patients died early postoperatively. Patients diagnosed with sarcoma had a significantly worse outcome than non-sarcoma patients (83.3 ± 15.2 % after 1 year, 31.3 ± 24.5 % after 5 years vs. 83.3 ± 15.2 % after 1 year, 0 ± 0 % after 5 years, *p* = 0.005).

**Conclusions:**

Malignancies with extension into cardiac structures or infiltration of great vessels can be resected with cardiopulmonary bypass support and tolerable risk. Carefully selected patients can undergo advanced operative procedures with an acceptable 1-year-survival, but only few patients achieved good long-term outcome.

## Background

Thoracic malignancies invading the mediastinum, heart or great vessels are rare and treatment including radical resection challenges oncological therapists. Primary mediastinal neoplasms without invasion into adjacent structures are generally of non-cardiac origin, such as thymoma, lymphoma or neurogenic tumors. Tumors that infiltrate directly into the heart, lung, aorta or vena cava are either primary cardiac malignancies or metastases of distant malignoma. The most common primary cardiac tumors are sarcoma (especially rhabdomyosarcoma or angiosarcoma) and lymphoma [1–6]. Once cardiac structures or great vessels are widely infiltrated, patients are merely treated in only a palliative setting due to an unresectable situation. Nevertheless, very few of these patients might qualify for surgery if local resection seems feasible. In these patients cardiopulmonary bypass (CPB) and cardioplegic cardiac arrest may be necessary in order to enable resection of infiltrated cardiac structures and subsequent reconstruction [7–15]. This interdisciplinary approach requires treatment at a cardiothoracic center and makes meticulous planning within a tumor board mandatory. Nevertheless, CPB-support for resection of advanced cardiothoracic malignancies increases the risk of bleeding from large wound surfaces and may promote a dissemination of tumor cells. Furthermore, immunological responses to extracorporeal circulation bear further risks like lung injury or organ dysfunction [16–19].

The clinical experience with these patients is very limited. Therefore, we report our ten-year single-center experience with patients suffering from thoracic malignoma who require the use of cardiopulmonary bypass during resection.

## Methods

**Fig. 1:**
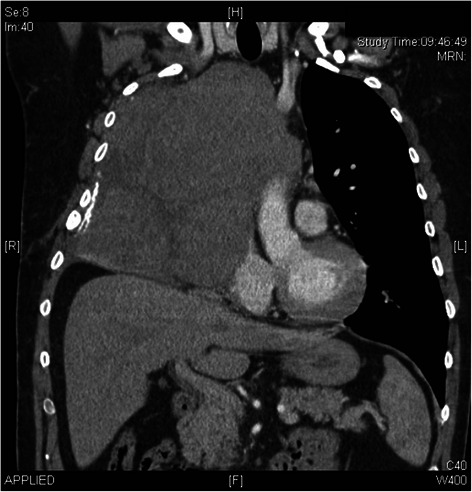
Preoperative computed tomography scan of patient 11. Carcinoid infiltrating right lung and Vena cava superior

Clinical data of patients that underwent surgery at our institution between 01/2002 and 05/2014 was retrospectively analyzed. Fifteen patients (12 female) with a mean age of 55 ± 15 years (range 24 to 80 years) were finally included. All patients had advanced thoracic malignancies and cardiopulmonary bypass support during resection. Two of them underwent combined cardiac surgery for coronary artery bypass grafting (CABG) in addition to tumor resection. All patients were treated in a context of interdisciplinary discussion including both local departments of cardiac and thoracic surgery. Preoperative routine staging included contrast enhanced ECG-gated computed tomography (CT) (Fig. 1) and magnet resonance imaging (MRI) of the chest. Clinical absence of extrathoracic metastasis or active malignant disease was a precondition for surgery. Five patients had diagnostic biopsy prior to operation. Intraoperative transesophageal echocardiography was performed in all patients. The site of cannulation for CPB was selected depending on the tumor localization. Conventional central bi-caval and aortic cannulation was performed in 9 patients, two-stage cannulation of the right atrium (RA) in 1 patient, peripheral cannulation of groin vessels in 5 patients with additional cannulation of internal jugular vein in 1 patient (patient #7). The diagnosis of a malignant tumor was made in 8 patients preoperatively. Final diagnosis was achieved by intraoperative instantaneous section and postoperative histology. Completeness of tumor resection was made by R classification as set by the American Joint Committee on Cancer [20]. All patients were finally debated in an interdisciplinary tumor board concerning postoperative therapies. Adjuvant chemotherapy was advised on an individual basis and was performed in 4 patients. Patient #6 denied adjuvant chemotherapy.

Pre-, intra- and postoperative data was retrospectively analyzed. End points were complications, overall survival and individual outcome. Early events are defined as in-hospital complications. Late events occurred after hospital discharge. After approval of the institutional review board, follow-up was obtained through contact with general physicians or with the patient or family directly. In accordance with the local ethic committee, the requirement of individual patient consent was waived because of the study’s retrospective design and the data collection from routine care. Mean follow-up time was 22 ± 30 months (range 4 to 112). Survival was estimated by use of Kaplan-Meier method. Survival curves were compared using log-rank test. Continuous variables are shown as mean ± standard deviation or as range; categorical data as percentage.

## Results

### Preoperative data

Eleven (8 female) patients were diagnosed with primary thoracic malignancies and 4 patients (3 female) with metastatic disease infiltrating cardiac structures and/or great vessels (Table 1). Patients with mediastinal metastases were free of local extrathoracic disease before cardiothoracic surgery. Primary tumors of metastatic disease were chondrosarcoma (left heel; infiltration of middle lobe, right lower lobe and left atrium (LA)), malign melanoma (infiltration of left lung (LL) and LA), osteosarcoma (left femur; infiltration of left lower lobe (LLL) and LA) and rectum carcinoma (infiltration of LL and LA).

**Table 1:** Patient characteristics, histological tumor diagnosis, localization, operative access, resected structures, R graduation, therapy aim and follow-up

Pts. No.	Sex	Age	Histological diagnosis and localization	Access	Resection	Induction, Aim	Follow-up (month)	Current status	R, Tumor recurrence
1	F	80	Thymoma, anterior mediastinum	Median sternotomy	Tumor anterior mediastinum	None, C	0.07	Dead	R0
2	F	35	Thymic b-cell Non-Hodgkin lymphoma, anterior mediastinum	Median sternotomy	Tumor anterior mediastinum	None, C	112.3	Alive	R1
3	F	54	Paraganglioma, posterior mediastinum infiltrating LA	Median sternotomy	*Ex situ* LA, posterior mediastinum, Tumor	None, P	0.67	Dead	R?
4	F	66	Intimal sarcoma, MPA and RVOT with tumor thrombus in RUL and RPA	Median sternotomy	MPA, RVOT, tumor thrombus	None, C	19	Dead	R0, lung metastasis
5	F	58	Chondrosarcoma metastasis, ML, partial RLL and LA	Right thoracotomy	ML, partial RLL, LA	Chemo, P	54.8	Alive	R0
6	F	49	Pleomorphic intimal sarcoma, MPA, LPA and LL	Median sternotomy	LL, MPA, LPA	None, C	21.3	Dead	R1, progressive recurrence in PA
7	M	62	Thymic carcinoma, VCS and portions within RA	Median sternotomy	Tumor, VCS	None, C	33.4	Dead	R1, unclear
8	F	57	Melanoma metastasis, LL and partial LA	Clamshell	LL, partial LA	None, C	45.4	Alive	R0, CNS metastasis
9	F	65	Pleomorphic rhabdomyosarcoma, LA	Median sternotomy	Tumor, partial LA	None, C	7.2	Dead	R1, local recurrence
10	F	24	Osteosarcoma metastasis, LLL and partial LA	Left thoracotomy	LLL, partial LA	Chemo, C	25.8	Dead	Questionable R1, CNS metastasis
11	M	45	Carcinoid, RL, Aorta, RA and VCS	Clamshell	RL, VCS	Chemo, P	0.00	Dead	R?
12	F	77	Paraganglioma, Aorta posterior mediastinum	Median sternotomy	Tumor, Aortic adventitia	None, C	4.7	Alive	R0
13	M	40	Undifferentiated sarcoma (NOS), RL, MPA and LPA	Median sternotomy, right thoracotomy	RL, MPA + LPA	Chemo, C	4.3	Alive	R0
14	F	46	NSCLC (adenocarcinoma) RL infiltrating LV and MV	Hemi-clamshell	RL, MV + posterior-med. PM	Chemo, C	3.53	Dead	R1, lung metastasis
15	F	61	Rectum carcinoma metastasis, LL, LA	Clamshell	LL, partial LA	Chemo, C	3.2	Alive	R0

*Pts.* patients, *LA* left atrium, *RA* right atrium, *MPA* main pulmonary artery, *RPA* right pulmonary artery, *LPA* left pulmonary artery, *RVOT* right ventricular outflow tract, *ML* middle lobe, *RUL* right upper lobe, *RLL* right lower lobe, *LLL* left lower lobe, *LL* left lung, *RL* right lung, *VCS* vena cava superior, *CNS* central nervous system, *C* curative, *P* palliative, *PM* papillary muscle, *NSCLC* non small cell lung carcinoma, *MV* mitral valve, *PM* papillary muscle

Patients #1 and #9 were preoperatively diagnosed with coronary artery disease (CAD) and required simultaneous CABG. Patient characteristics, histological tumor diagnosis, operative access, resected structures, R- status, intent of resection approach and follow-up are shown in Table 1.

### Operative data

Operation time was 303 ± 182 min (range 140 to 800 min) and CPB time 151 ± 99 min (range 75 to 405 min). Six patients required aortic cross-clamping (53 ± 65 min; range 36 to 173 min). Intraoperative hypothermic levels were set at 31.8 ± 3.8° Celsius (range 20 to 34° Celsius). No patient required total circulatory arrest. Four patients received auto-transfusion with cell-saver blood. Blood cell transfusion requirements measured 3433 ± 5743 ml (range 0 to 19,000 ml). Surgical access to the mediastinum and thoracic cavity was guaranteed through median sternotomy (*n* = 8), clamshell-incision (*n* = 4) and lateral thoracotomy (*n* = 3). Extended pulmonary resections were necessary in 8 cases. Circulatory support by IABP was performed in patient #1, who simultaneously underwent CABG, and in patients #3 and #4 due to cardiac low output.

**Fig. 2:**
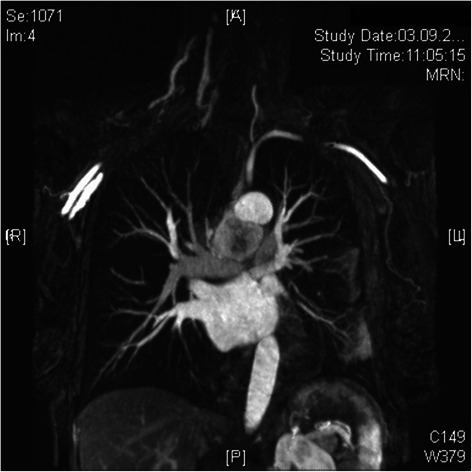
Preoperative magnetic resonance scan of patient 3. Paraganglioma of the posterior mediastinum infiltrating left atrium completely

**Fig. 3:**
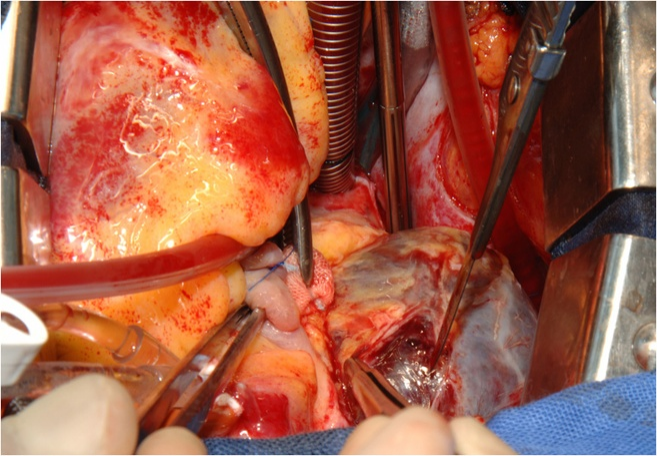
Intraoperative photograph of patient 3. Left atrium is completely infiltrated by paraganglioma

**Fig. 4:**
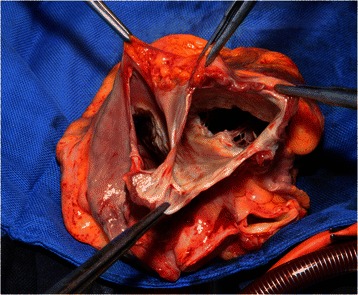
Intraoperative photograph of patient 3. Left atrium is resected *ex vivo* after temporary heart explantation and prepared for re-implantation

Patient #3 suffered from malignant paraganglioma located in the posterior mediastinum with extended infiltration into the left atrium. Complete resection required *ex situ* tumor removal of the heart with subsequent cardiac auto-transplantation (Figs. 2 and 3): After institution of CPB following bi-caval and ascending aortic cannulation, the heart was explanted. The tumor mass infiltrating the entire left atrial wall was resected respecting a 5 mm margin alongside the atrioventricular junction (Figs. 3 and 4). After resection of the extracardial tumor portion up to the vertebral column, a left neo-atrium was created using glutaraldehyd preserved autologous pericardium anastomosed to both pulmonary vein cuffs. Then, the explanted heart was re-implanted suturing the left atrial wall margin to the neoatrium. After auto-transplantation and weaning from CPB with support of an IABP the thorax was left open and covered with a Goretex membrane. The patient was transferred to ICU. After subsequent ECMO implantation the patient died due to multi organ failure on 19^th^ POD.

Complete resection (R0) was pathologically confirmed in 6 cases, 4 patients had tumor-positive resection margins microscopically (R1). R-status remained unclear in 3 patients.

### Postoperative course and outcome

One patient (#11) died intraoperatively due to right hart failure. Two patients died early postoperatively (within 30 days) due to multiorgan failure at POD 1 and 19. All other patients (*n* = 12) survived the early postoperative course. Overall ventilation time was 2.5 ± 4.9 days (range 0 to 20 d), in hospital survivors 1.3 ± 0.9 days (range 1 to 4 d) respectively, Overall ICU stay was 4.6 ± 5.4 days (range 1 to 20 d), in hospital survivors 3.3 ± 2.5 days (range 1 to 4 d) respectively.

Postoperative early and late morbidity is shown in Table 2. Complications requiring surgical intervention were recurrent pleural effusion (2 patients) and development of hemothorax (1 patient). Two patients received adjuvant chemotherapy, one patient postoperative mediastinal irradiation. Four patients underwent combined radio- and chemotherapy.

**Table 2:** Early and late morbidity and mortality

Patient No.	Early complications/therapy	Late complications/therapy	Cause of death
1	in-hospital death		MOF
2	superficial WHD	R-CHOEP chemo, Rx	
3	IABP, ECMO, bleeding, in-hospital death		MOF
4		lung metastasis, chemo	Pulmonary failure
5	right PLE	chemo	
6	haematothorax ➔ VATS	Rx not possible, palliative chemo declined by patient	cardio-pulmonary failure
7	PD, PLE	Rx	unclear
8		CNS metastasis, hypothyreoidism under chemo, radiotherapy	
9		early recurrent tumor in LA, palliative re-operation with tumor mass reduction	cardiac failure
10		adjuvant chemo – stop due toxicity, diagnostic partial pulmonary resections, CNS metastasis with bleeding, palliative radiotherapy	CNS death
11	in-hospital death		intraoperative right heart failure
12	intraoperative endograft implantation for Aortic descendens rupture	N. recurrens paresis	
13		chemo, Rx	
14		lung metastasis 2 month after discharge	tumor
15			

*MOF* multi organ failure, *WHD* wound healing disorder, *IABP* intra-aortic balloon pump, *ECMO* extracorporeal membrane oxygenation, *PLE* pleura effusion, *VATS* video-assisted thoracic surgery, *PD* postoperative delirium, *CNS* central nervous system

Local recurrence of disease emerged in 2 patients. One of these (patient #6) underwent re-surgery within 1 year after primary therapy with R1 resection status. Salvage surgery was performed in a palliative intent to reduce a left atrial tumor mass. Nevertheless, that patient died due to left atrial tumor occlusion and consecutive cardiac output failure during the further postoperative course. 3 patients developed lung and brain metastases and received individual oncological therapy.

**Fig. 5:**
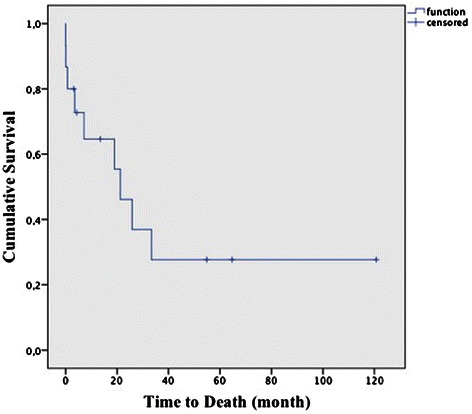
Actuarial overall survival after tumor resection of all patients (*n* = 15)

Overall actuarial survival was 65 ± 13 % at 1 year and 28 ± 13 % at 5 years (Fig. 5a). Mean Follow- up was 22 ± 30 months (range 4 to 112), six patients are alive at the end of follow-up. Survival in patients with sarcoma histology (80 ± 18 % after 1 year, 20 ± 18 % after 5 years) was found significantly poorer than survival in patients with other dignities (53 ± 17 % after 1 year, 36 ± 19 % after 5 years, *p* = 0.006).

## Discussion

There are only few reports of patients undergoing resection of thoracic malignancies with locally advanced invasion into the heart and great central vessels. Molina at al. described an incidence of primary cardiac tumors of 0.002 % in an autopsy series [3]. 75 % of primary cardiac tumors are benign with 2/3 of them being diagnosed as myxoma. One quarter of patients suffers from tumors with sarcoma histology, merely angiosarcoma or intimal sarcoma of great vessels. Unfortunately, most patients were initially found with locally advanced disease and infiltration of adjacent vital structures. Therefore, therapies remained mainly palliative employing conservative chemo- or radiotherapy. Since the early 1990s, first reports favored surgical treatment of malignancies infiltrating cardiac structures by showing improved survival compared to conservative treatment in selected patients [4, 5]. With further improvement of perioperative management including ventilation strategies, circulatory assistance and adjuvant procedures, curative therapy appeared achievable. However, if cardiac structures or great vessels are infiltrated, R0 resection remains challenging and extracorporeal circulation is often required.

Nevertheless, CPB implies several risks. The need of full heparinization may cause excessive bleeding from the surgical field, especially if additional extended lung resection is required. We observed prolonged postsurgical bleeding in 3 of our patients, but only one (patient #6) required redo surgery by videothoracoscopy after development of a hemothorax. Therefore bleeding complications were relatively low in our cohort. Another reported risk of CPB is the possibility of intraoperative tumor spilling, probably supported by venous drainage into the reservoir and re-circulation within the open system of CPB. Four of our patients developed distant metastases, 2 of them after initially complete (R0) resection. One of these suffered from malign melanoma, which is principally associated with a high risk of systemic tumor spread. Unless we did not observe an association with CPB-use and early development of distant metastasis, the avoidance of re-draining suctioned blood from the surgical field into the corporal system might reduce the risk of cell spilling in malignant surgery.

Third, immune modulatory effects are well described and remain a risk for patients undergoing surgery with use of extracorporeal circulation. In addition, patients with lung disease bear a greater risk for pulmonary failure and long-term ventilation after surgery involving CPB [18, 19].

Our cohort includes 8 patients that underwent partial lung resection (*n* = 2) or even pneumonectomy (*n* = 6). Only one of them (patient #11) died intra-operatively due to right heart failure. The other 7 patients showed a median survival of 20 months (range 3 to 55 months) without developing lung or circulatory failure within the early postoperative course. Despite the reported risk for lung failure that can be caused by CPB, none of our patients required tracheotomy or long-term ventilation therapy with an overall ventilation time of 2.5 ± 4.9 days (range 0 to 20), respectively 1.3 ± 0.9 days (range 1 to 4) in hospital survivors. These findings underline the technical feasibility of CPB-usage in operations that include partial or total lung resection without encountering an increased risk of pulmonary complications during the postoperative course.

Regarding the low incidence of tumors with only invading the heart and/or great vessels, the cohort we present here is quite heterogenic. In addition, extended cardiothoracic surgery with extracorporeal bypass support represents a high-risk therapeutic approach. Therefore, careful patient selection and intense interdisciplinary discussion within a tumor-board is necessary to point out candidates for surgery. Due to the tumor site and close proximity to vital structures, even surgical salvage procedures can be indicated to prevent secondary complications e.g. tumor-embolism or cardiac tamponade. Such a “palliative” strategy was aimed in 3 of our cases (patients #3, #7 and #11). Nevertheless, all patients were free of extrathoracic malignant foci at time of primary decision.

Preoperative diagnosis of the very malignancy was made in about half of our patients, of whom 5 underwent biopsy. Hence, more interventional or open biopsy prior to operation would have supported multi-modal therapeutic strategies in respect of achieving complete tumor resection e.g. by neoadjuvant therapy, which could lead to tumor mass reduction. Four of our patients underwent systemic induction therapy before tumor resection. In all other patients surgery appeared advisable without induction therapy, either because of rapid clinical deterioration at the time of diagnosis or lack of knowledge of tumor dignity. An example for the former situation is patient #2 (male, 35 years) who initially presented with severe progressive dyspnea at time of first diagnosis. Imaging showed a massive mediastinal mass with compression of the atrial cavities and superior vena cava. In an interdisciplinary debate with respect to the patients’ decision, early surgical resection was indicated rather than primary chemotherapy, even though the latter would have been advised by current guidelines [21]. After macroscopically complete resection (R1, histological diagnosis: Non Hodgkin lymphoma) and immediate relief of his symptoms, chemo- and radiotherapy were administered postoperatively with a favourable outcome. In individual cases, simultaneous functional cardiac disease requiring surgery may influence therapeutic strategy: patient #1 with diagnosis of a thymoma would formally have required induction chemotherapy. However, that patient had to be operated urgently due to concomitant CAD with need of CABG. Therefore, the tumor board advised surgical treatment in favour of induction therapy.

We describe a cohort of patients with locally advanced disease and involvement of multiple organs or, with respect to the heart, multiple cavities. Therefore, intraoperative findings sometimes force to substantially modify the preoperative planned therapeutic strategy: patient #2 was initially planned for resection without CPB. However, due to intraoperative poor hemodynamics, initiation of CPB was required to unload the heart. Local findings in patient #3 necessitated *ex vivo* resection of the tumor mass from the heart and reconstruction of the left atrium as described by few other authors before [22, 23].

A general principle in oncological surgery is an en bloc, complete tumor removal. The same applies for patients with locally advanced malignancies, even though local technical and functional resectability may force the surgeon to tolerate minimal resection margins or even microscopic tumor residual R1). R0-status could be achieved in 7 of our patients, R1 was found postoperatively in 6 patients. Albeit a very heterogenic group of patients undergoes cardiothoracic surgery for tumor removal with bypass support, acceptable survival-rates can be observed. Park et al. reported a series of 10 patients with an overall median survival of 21.7 months (range 3.2 to 69 months) and 33.3 months (range 3.7 to 69 months) after surgery with incomplete resection in 7 cases. There was no perioperative death observed [12]. In another series of Vaporciyan et al. 19 patients were examined and showed overall 1- and 2-year survival rates of 65 and 45 %, respectively. The authors found incomplete resection in only 4 of their patients but initially included a relatively large collective (*n* = 8) of patients with simultaneous extrathoracic tumor. Survival in that “palliative” group was poorer but still reached 10.3 + − 8.6 months [8]. Comparable overall survival were reported by Wiebe et al. with focus on extended pulmonary resection requiring cardiopulmonary bypass support. Cumulative survival in their series was 62 % at 1 year and 53 % at 3 and 5 years, respectively. Patients with histology of sarcoma showed a better outcome than those with non-small cell carcinoma (62.5 % vs 33 % after 1 year) [7]. Analysis of our data showed an actuarial overall survival of 65 ± 13 % at 1 year and 28 ± 13 % at 5 years, which is comparable to other centers. Even though our series as well as literature reports comprise only few patients with rather heterogenic diseases survival differences depending on histology have been observed. In contrast to Wiebe et al., we found significant poorer survival in patients with sarcoma compared to non-sarcoma. However, Wiebes series contained many patients with non-small lung cancer whereas we also considered other mediastinal malignancies like thymoma, thymic carcinoma or carcinoid tumors with substantially better prognosis [7]. Sole analysis of patients with cardiac sarcoma was recently published by Li et al. In their series of 29 patients, they reported an overall median survival of 17 months, expectably with a significantly better survival for R0 resection status [24]. With regard to our single center results and overview of the current literature, resection of malignancies invasion into the heart or great vessels are extremely rare procedures. Main pathological entities are sarcomas, ensued by secondary tumors with metastatic lesions invading central mediastinal structures. Surgical resection with bypass support seems feasible with acceptable outcome even in patients with so called “palliative” intent. Complications occur occasionally during CPB-usage and where mainly bleeding events that were manageable by secondary revision. Even patients with incomplete tumor removal might profit in selected cases as well as those patients that are critically limited by mechanical tumor-associated symptoms. However, it is evident that consecutive interdisciplinary discussion to determine oncological therapy is strictly mandatory in all patients.

## Conclusions

In conclusion, our review of patients with need of cardiopulmonary bypass support for resection of central tumors shows that the procedure can be performed with tolerable risk and acceptable outcome in carefully selected patients. Certainly, survival is limited but would be presumably worse administering non-surgical therapies. Therefore, well-chosen patients might benefit from surgery even in a palliative intent. Nevertheless, meticulous weighing of conservative and surgical treatment options within a tumor board is mandatory. Thus, definite preoperative diagnosis is of paramount importance and interventional or open biopsy should be performed if imaging not suffices. Patients should be informed that curative resection might be uncertain, in particular in patients suffering from sarcoma.

## Abbreviations

C: Curative
CABG: Coronary artery bypass grafting
CAD: Coronary artery disease
CNS: Central nervous system
CPB: Cardiopulmonary bypass
CT: Computed tomography
ECMO: Extracorporeal membrane oxygenation
IABP: Intra-aortic balloon pump
ICU: Intensive care unit
LA: Left atrium
LL: Left lung
LLL: Left lower lobe
LPA: Left pulmonary artery
ML: Middle lobe
MOF: Multi organ failure
MPA: Main pulmonary artery
MRI: Magnet resonance imaging
NOS: Undifferentiated sarcoma, not otherwise specified
P: Palliative
PD: Postoperative delirium
PLE: Pleura effusion
RA: Right atrium
RL: Right lung
RLL: Right lower lobe
RPA: Right pulmonary artery
RUL: Right upper lobe
RVOT: Right ventricular outflow tract
VATS: Video-assisted thoracic surgery
VCS: Vena cava superior
WHD: Wound healing disorder
